# On Iodine as a Deodoriser and Disinfectant

**Published:** 1864-04

**Authors:** B. W. Richardson

**Affiliations:** London


### ON IODINE AS A DEODORISER AND DISINFECTANT.

By Dr. B. W. Richardson, M.A., London.

The following is from a report of papers read at the late meeting of the British Association at Newcastle:—

Dr. Richardson made a short observation in sub-Section D on Thursday, August 27, "On the Application of Iodine for Disinfecting and Deodorizing Purposes." The iodine should be placed in a common chip-box, such as is employed by pharmaceutists, the lid of the box being replaced by a covering of "leno," or the iodine may be placed in the ornamental vases on the mantel-shelf of a room. The smell of iodine could thus be communicated to the air of an apartment, and air so purified was not only fresh and agreeable to the sense of smell, but any organic matters present in it were destroyed. In extreme cases the iodine should be placed on a dish or plate, and the heat of a candle being applied beneath, the iodine was volatilized, and a room was quickly purified. Dr. Richardson said that in cases of small-pox a knowledge of the facts he had named was most valuable. In rooms occupied by sufferers from this painful disease, organic matters floated largely in the air, rendering the air most offensive. He (Dr. Richardson) had succeeded, in all cases, in rendering such air inodorous by the volatilization of iodine. He had also observed the singular fact, that when the air was greatly charged with organic materials, the smell of the iodine was for a long time imperceptible, so that in truth the iodine method of purification was also a ready and practical test of the purity of an air. Dr. Richardson thought the iodine plan was quite as effective as the liberation of free ozone—it was, indeed, in principle the same, and was so simple that every person could employ it.

Dr. Wood said that the iodine produced the effects named by Dr. Richardson in one of two ways—it either destroyed the organic compounds by combining with hydrogen, and forming hydriodic acid, or it might be that in the chemical changes which occurred ozone was formed. In either case there was unquestionably a rapid and effective process of oxidation, the results being the same as occurred with chlorine to a considerable extent, and in a manner more easy and manageable.—*Medical Times and Gazette, Sept.* 26, 1863, *p.* 334.

